# Analysis of the Internal Marketing Dimensions in Social Economy Organizations: Study Applied to Co-operativism in Ecuador

**DOI:** 10.3389/fpsyg.2020.580673

**Published:** 2020-09-25

**Authors:** Francisco González Santa Cruz, Nelly Moreira Mero, María Iliana Loor Alcívar, Amalia Hidalgo Fernández

**Affiliations:** ^1^ Faculty of Law and Business Administration, University of Córdoba, Córdoba, Spain; ^2^ Faculty of Accounting and Auditing, University Eloy Alfaro of Manabí, Manta, Ecuador

**Keywords:** internal marketing dimensions, co-creation of value, measuring scale, co-operatives, Ecuador

## Abstract

Internal marketing involves the development of organizational strategies that promote the welfare of the employees who, in turn, play a transcendental role in achieving institutional goals. Ecuadoran co-operativism lacks relevant studies of this construct and, because of this, this research intends to analyze the dimensions of internal marketing, through the validation of a measuring instrument that has been adapted to this sector of the social economy, in a developing country. The methodology is based on the completion of field work, where a structured questionnaire for a representative sample of 2,499 employees and officers of the operatives of Ecuador was applied. The suitability of the scale was determined through the means of a model of Ecuadoran structural equations. The results show that internal marketing is a multi-dimensional construct and it can be measured in six dimensions: Identify Value Exchange (IVE), Internal Market Segmentation (IMS), Internal Communication (IC), Management Concern (MC), Training (TR), and Work/Family Balance (WFB).

## Introduction

A globalized economy requires all the businesses, including co-operatives that are constantly restructuring to adapt to changes in the environment (Puusa et al., 2016); therefore, direct their efforts to human talent in order to improve their internal management. Taking into account that human capital is a resource that is difficult to imitate due to competition, it has thus become an important factor for organizational success (Teixeira, 2014). In this sense, Bailey et al. (2016) confirm that internal marketing should be considered as a strategy where the employees are involved in the goals of the organization and, thus, feel satisfied with the work done. For their part, González et al. (2016) consider that a committed employee actively contributes to the achievement of institutional goals. In this sense, research indicates that internal marketing is a serious alternative for increasing the employee’s commitment to the organization and the subsequent satisfaction of the customer (Ali, 2016; Therasa et al., 2017).

Currently, internal marketing is a concept that continues to evolve to show the importance that relationships between employees and the organization have (Braimah, 2016; Park and Tran, 2018; Vieira-dos Santos and Goncalves, 2018). In this case, diverse authors (among others, Ruiz-Alba, 2013; Ruizalba et al., 2015; Mainardes et al., 2019) have defined it as the effort by the company to know, analyze, understand, and respond to the needs of its internal customers (employees). With reference to this, Papasolomou and Vrontis (2006) consider that investing in employee motivation is undoubtedly an essential requirement in the search for competitive advantages in businesses. As such, the essence of co-operatives, as social economy businesses, is related to the internal marketing dimensions given that it is based on balance among material, financial, and human resources (Fernández et al., 2018). Similarly, Ahmed and Rafiq (2003) indicated that internal marketing helps the development and maintenance of the inter-functional relationships among employees who, as internal customers, are involved in the co-creation of value, which is used to attain success within the organizations.

In this context, the International Cooperative Alliance has significantly contributed to the bilateral processes of knowledge transfer through inter-organizational relationships on a local, national, and international level, leading to the institutional strengthening of these co-operatives. For their part, the Swiss Co-operation Agency has driven the initiatives for strategy inclusion in the Ecuadoran business world, with internal marketing being one of them. However, these external contributions have not reached the management of all the co-operatives of this Latin American country. This leads us to the need to study the dimensions of internal marketing, in relation to the policies applied in this sector for the motivation and involvement of their employees. In any case, every country and global sector has a series of socio-economic and cultural activities and characteristics that make them different (Huang and Rundle-Thiele, 2014).

Accordingly, the co-operative sector faces diverse problems, such as inequality regarding incentives, excessive controls, bureaucracy, and strong competition, even with commercial companies. Therefore, the challenge that goes with this problem is focused on creating strategies that allow for taking advantage of strengths derived from its co-operative principles for the co-creation of value. This should guarantee continuity and expansion in the market (Ferraz et al., 2018). Hence, this study aims to provide a measurement tool that fits the specific needs and characteristics of the cooperative sector, considering the complexity of the study of this sector, which encompasses multiple activities. Mainly financial (savings and credit cooperatives) and non-financial (housing cooperatives, services, production, and consumption) stand out in Ecuador. This instrument could enable these organizations to know the perception of their employees, regarding the strategies that are implemented for the management of human capital and undertake improvement actions that allow the co-creation of value and organizational sustainability.

Therefore, the study of internal marketing in Ecuadorian cooperatives has been considered appropriate, since they represent a significant part of the social economy, especially in developing countries. Thus, the main objective of this research is to analyze and validate, if seen fit, the dimensions of internal marketing through the application of a measuring instrument adapted to the cooperative sector. With this starting point, this study uses the Jaworski and Kohli (1993) and Lings (1999) model, with this being the one which best supports the compliance of the aim of the investigation, due to having a synergy connection between the internal market (business) and the internal customer (employee). The suggested scale corresponds to the adaptation of the internal marketing dimensions considered by various authors (among others, Ruizalba et al., 2014, 2015; Kim et al., 2016; Cerqueira et al., 2018).

This article is structured into five main sections and begins with the literature review, which consists of the scientific basis before this study. Secondly, there is the methodology of the research based on the field work and the measuring scale. In the following, the results will be presented through a model of structural equations and their discussion. Finally, the conclusions are suggested, as well as the limitations of the study and future lines of research.

## Literature Review

### Internal Marketing

Strategic management is a significant element that leads to the success of an organization, a process which should be focused on employees, who, with their intellectual capacity, skills, abilities, and motivations, provide jobs that are prone to achieving institutional goals (Abzari et al., 2011). The approaches presented by the conceptualizations of internal marketing also determine its influence on the external client, since an adequate working climate, where the wishes and needs of employees are considered, implies a better performance in their work activities. This, in turn, significantly influences external customer satisfaction (Lizote et al., 2019).

Internal marketing has its origin in the work of Berry et al. (1976), although this construct continues to be in constant evolution, allowing for advances in its conceptualization. In this way, Lings (1999) proposes that, in addition to the employee knowing the business’ information, the officers should be interested in the employees’ needs and wishes. From this point of view, Ahmed and Rafiq (2002) define internal marketing as the planned effort of motivating employees through marketing techniques, to implant and integrate business strategies aimed at the customer. In addition, Anosike and Ahmed (2009) indicate that the exchange of ideas between an employer and an employee contributes to achieving common benefits. For their part, Cerqueira et al. (2018) propose treating employees as internal customers and their activities as internal products, which satisfy the needs and wishes of external customers.

As such, the adoption of internal marketing allows for the business to align its strategies with the needs of the internal customer in order to introduce benefits to the organization (Gounaris et al., 2010). In this way, institutions that apply this management philosophy ensure the efficiency of the service provided by the employees, which would help improve the satisfaction of the external customer (Barzoki and Ghujali, 2013; Jaeger et al., 2016; Lizote et al., 2019). In this respect, Oliveira and Pataco (2017) confirm that motivated and loyal employees have more positive attitudes toward their businesses and their management, which leads to an increase in income. In this context, the internal marketing approach is basically aimed at all the interactive activities within the institutions, with the purpose of providing a quality work environment (Zebal, 2018). Because of this, the understanding of the dimensions of this construct allows for identifying the dimension that most contributes to the co-creation of value, given that internal marketing influences attitudes and behavior of the internal customer to transmit it, by doing so, to the external customer (Dean et al., 2016; Chiu et al., 2019).

The boom that has occurred in internal marketing in recent times comes from research that suggests diverse activities tending to know, analyze, and respond to the needs of employees, which has given way to the dimensions of this construct (Ruizalba et al., 2015). In this case, the theoretical references regarding internal marketing models and their dimensions were proposed by the precursors in this construct (Parasuraman et al., 1985; Grönroos, 1990; Jaworski and Kohli, 1993; Lings, 1999; Rafiq and Ahmed, 2000; Bansal et al., 2001).

Based on this, the importance of the dimensions which cover internal marketing in the co-operatives of Ecuador is highlighted, considering that it generates co-creation of value to the service, which provides the institutions that have applied it; thus, it improves their results (Yu et al., 2018). This has determined a growing interest from the officers and those responsible in the dimensions of internal marketing, as a primordial factor within the good practices of internal management. Albassami et al. (2015) shows that it is not only a statement of intent, but that it also respects the rights of the workers, bearing in mind equality, no discrimination, a dignified job, conciliation between family and professional life, etc. As such, it is necessary to consider the aspects indicated beforehand to measure internal marketing, with the idea of developing a measuring instrument of this construct that can be considered an effective tool for the management when making strategic decisions (Park and Tran, 2018).

### Dimensions of Internal Marketing

The analysis of the internal marketing dimensions sifts a fundamental part of the overall management of a business given that, through its study and application, the employee’s satisfaction and the subsequent achievement of the same goals can be reached (Abzari et al., 2011). This is the reason why diverse studies (among others, Lings and Greenley, 2010; Ali, 2016; Braimah, 2016) have led to defining two important variables within internal marketing, with the first being the identification of the business as an internal market and, the second, the determination of the employees as internal customers. In this way, the dimensions of this construct are projected to highlight these characteristics (Foreman and Money, 1995; Lux et al., 1996). However, Parasuraman et al. (1985) and Grönroos (1990) propose a unidimensional model of internal marketing, considering only the macro-dimension of customer orientation. Despite this model having been used in current research (among others, Yildiz and Ali, 2017; Piha and Avlonitis, 2018), it has been the subject of criticism and restructuring due to the limitations that it provides in the real application of the internal marketing strategies in businesses of different sizes and in different activity sectors.

All of this has led to the development of models, which determine a multi-dimensional perspective of internal marketing. In this sense, Jaworski and Kohli (1993) and Lings (1999) distinguish three dimensions of this construct: (1) generation of internal intelligence, (2) Internal Communication (IC), and (3) response to internal intelligence, which, at the same time, is divided into six sub-dimensions. For their part, Rafiq and Ahmed (2000) complete a redesign of the one-dimensional model of Parasuraman et al. (1985) and Grönroos (1990), adding three new dimensions: (1) empowerment, (2) inter-functional co-ordination, and (3) communication, establishing a model of four dimensions. Afterward, Bansal et al. (2001) suggest a model of six internal marketing dimensions: (1) employment security, (2) extensive training, (3) generous rewards, (4) sharing information, (5) employee empowerment, and (6) reduced status distinction.

The application of the Jaworski and Kohli (1993) and Lings (1999) model in various studies has made it possible to determine the validity and consistency of its dimensions, albeit with a focus on mercantilist companies and in a geographical and cultural field different from that under study in this research (Ruizalba et al., 2015; Kim et al., 2016; Cerqueira et al., 2018).

More recent studies (among others, Ruizalba et al., 2014; Braimah, 2016; Cerqueira et al., 2018; Park and Tran, 2018; Mero et al., 2020) have redesigned the three dimension model of Jaworski and Kohli (1993) and Lings (1999), considering the initial sub-dimensions of the same as true first-class dimensions, which has led them to the suggestion of a much more complete and versatile model of six dimensions: Identify Value Exchange (IVE), Internal Market Segmentation (IMS), IC, Management Concern (MC), Training (TR), and work/family. For this reason, this research will use this vision of six internal marketing dimensions as a reference; given its great adaptability to different socio-economic environments which, in this empirical study, applies to Ecuador’s co-operativism, considered a developing country.

Using this model, it should be indicated that the IVE dimensions’ job is to collect information about the internal market, so that what the employees hope to receive from the business is known, as well as the benefits that they are going to give to the business. This component occurs when hiring staff, when the values of new staff are identified, as well as those of the organization, so as to know if they share the same philosophy (Lings, 2004). On the other hand, the IMS dimension, involves the separation of functions which each employee of the organization performs, considering aspects of macro-segmentation (socio-demographic aspects) and micro-segmentation (attitude, behavior, etc.), all of this at diverse levels of management (Gounaris, 2006).

For its part, the IC dimension is essential in all the inter-personal relationships that help the development of activities through the exchange of information between the leader and the employees (Chiu et al., 2019). In this sense, Ahmed and Rafiq (2002) show that communication should be extended to different levels of the organization, to be able to influence the behavior of all the employees. As such, the improvement of IC could offer unique capacities for a significant performance in the target market (Danso et al., 2017). MC, as an internal marketing dimension, suggests that supervisors should worry about knowing the present and future expectations that workers have (Ruizalba et al., 2014); disclosing good management practices to motivate employees in the performance of their work (Chareonwongsak, 2017). In relation to TR, this dimension looks to prepare employees in the development of abilities and capacities that the work position requires and, in turn, means an opportunity for its growth within the business (Abbas and Riaz, 2018).

Finally, in terms of the Work/Family Balance (WFB) dimension, Narteh (2012) considers the family as an interest group (stakeholder) of the business itself. And, in this case, businesses that want to keep their best employees have to favor the development of policies that allow for the harmonization of family and work life, as both are mutually sustained (Tang et al., 2020). These employees will feel understood by the business, when it develops strategies to reconcile their work and family life (Ruizalba et al., 2014; Solat et al., 2020).

In conclusion, the multi-dimensional vision of internal marketing has been analyzed in different empirical studies, mainly focused on the United States, Europe, and Asia, with excellent results that have allowed for the consolidation of internal marketing in these geographical areas (Clampitt and Downs, 1993; Back et al., 2011; Hoque et al., 2018), but there is an important deficit of empirical research regarding this construct in Latin America.

## Methodology

### Survey Design

The aim of this study is to analyze the dimensions of internal marketing, through the validation of a measuring instrument applicable to co-operativism in Ecuador. To achieve this, a preliminary list of measuring items was suggested; this was generated after a review of the literature related to the internal marketing dimensions. In the adaptation phase of the questionnaire, a review with Ecuadoran specialists involved with the popular and solidary economy sector (referred to in this way in the Latin American country) was carried out and used to discard errors in terms and understanding. Afterward, a pre-test was given to 40 participants, to detect possible deviations and errors directly in the field being studied.

The questionnaires have been prepared from two points of view, the administrators that represent the organization and the employees as internal customers. The structure of the questionnaire consists of open and closed questions, regarding aspects such as work and socio-demographic details. For their part, these items of internal marketing dimensions are measured using a Likert scale of five points, giving the surveyees the options that range between (1 = completely disagree and 5 = completely agree).

### Data Collection

The procedure of the field work consisted of a survey *in situ* with staff that works in the co-operatives. This questionnaire was applied to a team of surveyors from the Eloy Alfaro de Manabí Lay University (Ecuador). Initially, the purpose of the study was explained to the administrators of every co-operating party, requesting the respective permission of the employees. The questionnaires were designed in Spanish and they were complemented under the presence of surveyors, with the aim of responding to any question that may arise. The field work was done between the months of January and April 2018. The rejection rate was very low and it was not related to any specific variable. The length of the survey never lasted more than 15 min, in any case.

### Sample and Sampling Error

The population of this study includes administrators and employees of financial and non-financial co-operatives in Ecuador. As such, a stratified sampling was carried out which obtained 2,499 surveys. The co-operatives in Ecuador are classified in financials, who are additionally subdivided into segments from 1 to 5 (in terms of the figure of assets) and into non-financials, which are grouped by their branch of activity, with these being services, housing, production, and consumption (Superintendencia de Economía Popular y Solidaria (SEPS), 2017). The sample is distributed among 1,414 women and 1,085 men. The ages range from 18 to 60. A sample error of ±1.89% was considered for a confidence level of 95%.

### Data Analysis

Once the field work was finished, the verification of the data was undertaken, removing all those questionnaires that present absent values in any item. The SSPS v.23 software was used, where the descriptions for each one of the elements was calculated, studying its asymmetry, together with normality, on a univariant level as well as on a multi-variant one. Secondly, an exploratory factorial analysis and another confirmatory one were carried out, with a structural equations model to study the validity of the scale, using Amos Graphics v23 software. For the development of the research, a quantitative approach was applied, which was based on a deductive plan to look for mechanisms to confirm the viability of the instruments used.

## Results

### Design of the Measuring Scale

The theoretical analysis regarding internal marketing has allowed for the definition of this construct with its respective questions that were applied in the adaptation of the questionnaire, in a way which contributes with the information needed to achieve the aim of the study. The structure of the questionnaire consists of aspects related to socio-demographic data, such as age, sex, education, marital status, and children; as well as those related to work position and contract type, working day, length of service, role, and pay, among others. This information allows us to identify the organizational structure of Ecuadoran co-operatives. On the other hand, the dimensions of internal marketing are based on the current re-design of the Jaworski and Kohli (1993) and Lings (1999) models, based on six dimensions: IVE (four items), IMS (three items), IC (four items), MC (five items), TR (three items), and WFB (three items).

### Validity of the Measuring Scale

In the exploratory factor analysis, the method of main components with varimax rotation (maximum variance) was applied, which determined that all the common factors are greater than one, thus considering that they are adequate with the variables. The viability of the factorial analysis was evaluated with the following criteria: the original correlations presented a large number of correlations (88.90% with a value greater than 0.30), Bartlett’s test for sphericity showed that the variables were not independent [*χ* ^2^(231) = 11,114.48, *p* < 0.001]. The Kaiser-Meyer-Olkin (KMO) measure of appropriate sampling obtained a value of 0.893, being considered to be adequate. All the values of the Measures of Sampling Adequacy (MSA) were found to be above 0.88.

In the varimax rotation (Table 1), the criteria used for assigning an item to the factor was that which presented a factorial load greater than 0.30, which explains 72.31% of the total variance, determining six factors in this way. The first factor explains 15.63% of the total variance and has high and positive correlations with items 1–4; therefore, we call this first factor or dimension IVE. The second factor explains 12.37% of the total variance and has a positive correlation with items 5–7; identifying this factor as IMS. The third factor represents 11.61% of the total variance, with a positive correlation with items 8–11; which we refer to as IC. The fourth factor which explains 11.38% of the total variance has a positive correlation with items 12–16; which is known as MC. The fifth factor explains 11.32% of the total variance, having a positive correlation with items 17–19; and which we call TR. Finally, the sixth factor represents 10% of the total variance and has a positive correlation with items 20–22; this factor or dimension is known as WFB.

**Table 1:** Exploratory factor analysis internal marketing questionnaire.

	Dimension					
	IVE	IMS	IC	MC	TR	WFB
IM1 Your employees needs.	0.620					
IM2 Meetings between manager and employee	0.814					
IM3 Inquiry of expectations	0.809					
IM4 Prove labor satisfaction	0.658					
IM5 Well defined groups		0.774				
IM6 Design of socialized politics		0.610				
IM7 Equal opportunities		0.551				
IM8 Interest in management			0.521			
IM9 Communication of unemployment			0.472			
IM10 Permanent attention to employee			0.724			
IM11Direct information from management			0.561			
IM12 Inversion of resources on employees				0.701		
IM13 Ones necessities creates politics				0.725		
IM14 Management mediator of conflict				0.740		
IM15 Personalized attention toward employee				0.590		
IM16 Management compression				0.559		
IM17 Communication about new changes					0.779	
IM18 Permanent training					0.724	
IM19 Training for new positions					0.801	
IM20 Compassion toward family necessities						0.789
IM21 Support of work/family relationship						0.853
IM22 Encourages an equilibrium between work and family						0.684
Eigen values	8.16	2.61	1.87	1.35	1.19	1.09
% Variance explained	15.63	12.37	11.61	11.38	11.32	10.00
% Variance explained cumulative	15.63	28.00	39.61	50.99	62.31	72.31

Source: Own elaboration.

### Confirmatory Factor Analysis

**Figure 1:**
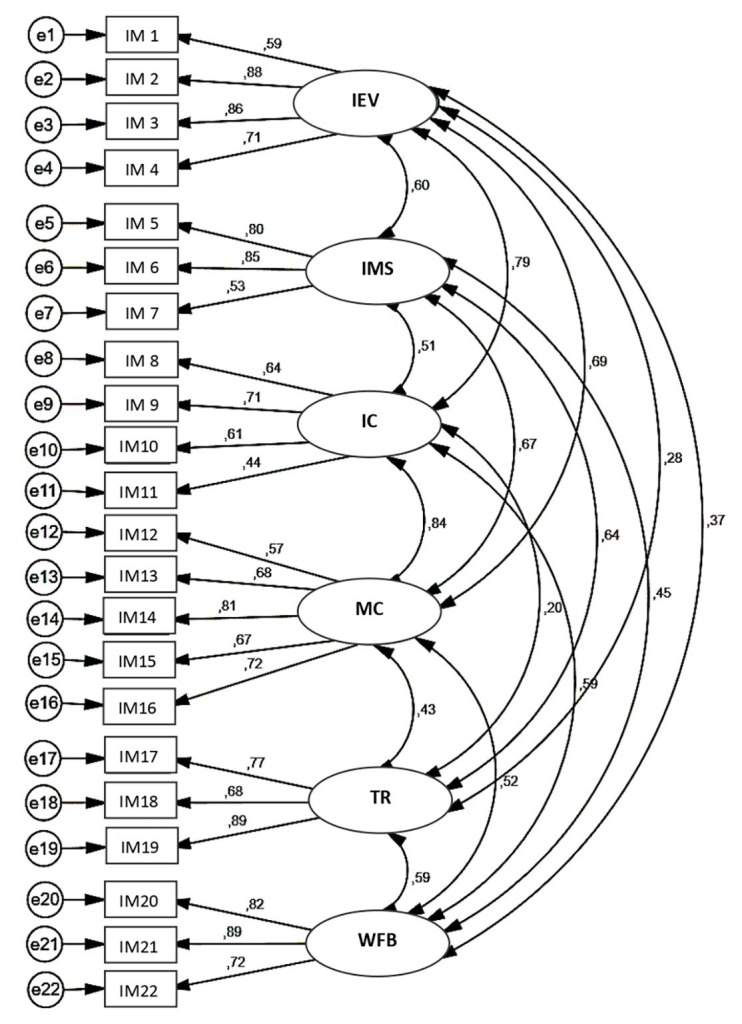
Estimation of structural model internal marketing.

The previous results of the exploratory analysis confirm the appropriateness of the measuring scale. Additionally, a confirmatory analysis with a model of structural equations and the application of the extraction method of maximum plausibility was carried out. All of this allows for the discovery of the convergent validity of the instrument proposed in the exploratory factor analysis, whose structure consists of six dimensions and 22 indicators in total. The results obtained were statistically significant (*p* < 0.05) and the factorial loads presented values greater than 0.5 by which we can indicate that all of the indicators satisfactorily saturate each latent variable. Definitively, it can be confirmed that the proposed model regarding the factorial structure of the scale is consistent (Figure 1).

For the analysis of the model’s Goodness of Fit Indexes (GFIs), comparative fit statistics were used, such as: Comparative Fit Index (CFI), Tucker Lewis Index (TLI), and Normed Fit Index (NFI). In this way as well, the unhurried fits GFI, Adjusted Goodness of Fit Index (AGFI), and Root Mean Square Error of Approximation (RMSEA). A confidence interval of 90% was considered. The results of the CFI, TLI, and NFI fit indexes show values greater than 0.96. For their part, the GFI and AGFI indexes presented values greater than 0.97 and the value obtained from the RMSEA is 0.048 (Table 2). These results indicate that the fit of the initial theoretical scale model is high.

**Table 2:** Goodness of fit statistics confirmatory factor analysis (CFA).

	*χ* ^2^(gl)	*p*	*χ* ^2^/gl	GFI	AGFI	CFI	NFI	TLI	RMSEA (I.C. 90%)
Total	2679.14 (194)	<0.001	13.80	0.97	0.97	0.97	0.96	0.97	0.048 (0.038–0.063)
Subsample 1	2121.47 (194)	<0.001	10.90	0.96	0.96	0.96	0.95	0.95	0.053 (0.044–0.074)
Subsample 2	2073.53 (194)	<0.001	10.70	0.94	0.96	0.95	0.94	0.95	0.052 (0.044–0.073)

Source: Own elaboration.

With respect to the reliability of the scale, Cronbach’s alpha coefficient was used which determines the internal consistency of the items of each of the dimensions. Bryman (2004) and Sekaran and Bougie (2010) coincide that Cronbach’s alpha coefficient, with an average of 0.5–0.7, is a moderate and acceptable level for research in social sciences. Thus, the greater the coefficient, the better the reliability of the scale will be (IVE = 0.921, IMS = 0.899, IC = 0.914, MC = 0.865, TR = 0.876, WFB = 0.899), which indicates a very high reliability.

## Discussion

Internal marketing originated from the need to improve the quality of services offered to external clients and through motivation, satisfaction, and the commitment of the employees, knowing that an employee who is satisfied and integrated in the business project increases the organization’s productivity and income (Mishra, 2018). This determines a strong relationship between employees and the orientation to the internal market with the development of competitive advantages for organizations. Employees are considered as unique assets, difficult to copy or imitate by competition and so, the orientation to the internal market involves indirectly influencing the perception of the external customer (Mainardes et al., 2019).

In this context, the literature review leads us to the analysis of the possible multidimensionality of internal marketing. Recent studies show such characterization in the empirical application of different theoretical models (Tang et al., 2020). With this background, the need to propose the adaptation of a scale that allows to measuring the relationship of each of the dimensions immersed in this construct and its adjustment to the particularities of the cooperative sector in developing countries is appropriate. Matters that seek to strengthen strategic management and provide tools that help to co-create value with what has been considered its brand of identification, that is to say, the co-operative principles and values in order to improve the labor climate in the organization (Dean et al., 2016; Dong et al., 2016; Kesen et al., 2017).

The study carried out allows for ensuring the validity and reliability of a scale to measure the dimensions of internal marketing in the co-operativism of Ecuador. In this way, the adaptation of the questionnaire used instruments that had already been applied to different empirical studies in different geographic environments. Among them is Ruizalba et al. (2014), which adopted and validated a questionnaire with six dimensions, through an empirical study applied to the hotel sector in Spain with a sample of 750 hotels. Similarly, Ali (2016) focused his work on five dimensions comprising 26 items, applied to 305 employees of the Arab Academy of Science, Technology and Maritime Transport of Egypt, obtaining significant results in all its dimensions. Sarker and Ashrafi (2018) applied a tool that analyzed seven dimensions of internal marketing, with a target population of 250 retail store employees in the city of Dhaka, Bangladesh, where the significant effect on employee satisfaction was evident. In turn, Ramos (2018) in his research on this construct, considered the employees of 39 financial institutions in the Philippines, who used a five-dimensional composite instrument, obtaining results appropriate to the objectives set.

More recently, Chiu et al. (2019) proposed a five-dimensional internal marketing measurement instrument, which was applied to workers from various sports centers in the city of Tapei, the results of which revealed adequate adjustments to the model. Tang et al. (2020) conducted a study based on an eight-dimensional scale, which reflects the perceptions of flight attendants of the most recognized airlines in the United States, in order to determine the effect of internal marketing on workers’ happiness, resulting in all dimensions having significant and positive relationships, with the exception of the compensation dimension.

This research context reflects the gap in the study of internal marketing in the sector of the social economy to which cooperatives belong, which are basically governed by universal principles focused on the human being. Reciprocity and balance are expected from these institutions in the actions employees-organization, in compliance with the principle of mutual assistance (Mero et al., 2020). It is for this reason that this quantitative study applied to employees and managers of Ecuadorian cooperatives has been raised, proving that it is appropriate and supports the internal marketing dimensions of the redesigned model of Jaworski and Kohli (1993) and Lings (1999), in its most recent and adapted version of six dimensions: IVE, IMS, IC, MC, TR, and WFB. It is important to indicate that the dimensions of this construct show bi-directionality (business-employee) in its structure, resulting in its easy adaptation for any type of institution of social economy, considering the few studies in this area of activity.

## Conclusion

Internal marketing is one of the primordial factors in the management of human talent and, for its development, strategies should be established which bear in mind the dimensions of this construct, with the purpose of satisfying the internal customer. Therefore, it shall provide a quality service that adapts to the characteristics and needs of the external customer, increasing the practices of co-creation of value through it. The results of this study provide a multi-dimensional instrument for measuring internal marketing, which seeks to strengthen the strategic management of the human resources of Ecuadorian cooperatives. Therefore, the study on the dimensions of this construct has its main application in the presentation of a scale that allows to measuring internal marketing, in such a way that they help these organizations to improve their relationship with employees. It should be considered that recent studies focused on this construct have been applied in purely mercantilist companies in United States, Europe, and Asia with satisfactory results. However, each area of application has different culture, values, norms, and ways of thinking and behaving; which justifies the necessary adaptation and validation of a questionnaire in accordance with the reality of Ecuadoran co-operativism in order to achieve the aims of the research.

The novelty of the study for the cooperative sector is highlighted, since they have different areas of activity and different market orientation than mercantilist companies. These institutions of the social economy have been identified by their co-operative values and principles, where the spirit of participation and collaboration for the search of socio-economic wellbeing and the satisfaction of their stakeholder are very important. With this idea, the measuring instrument for internal marketing for the co-operatives of this developing Latin American country has been obtained as a result of the empirical study carried out and its validation, demonstrating that internal marketing can be measured through six dimensions and 22 items, correlated among them, unlike the other studies where five dimensions have been proposed for the most part.

Definitively, the measuring scale provided in this research becomes an instrument that can be used in other sectors, financial ones as well as non-financial ones, given that the Ecuadoran co-operatives develop their activity in different sectorial environments, such as services, housing, production, and consumption, among others, and in very different volumes of business. As such, this research offers a tool for analyzing internal marketing, appropriately adaptable to different organizational realities.

Its application will allow leaders and managers to know the needs and expectations that employees have and, this way, improve the efficient management of human capital in their company. In addition, this implementation would also assist the specific control body of the Ecuadorian government, the Super intendency of Popular and Solidarity Economy, responsible for its promotion and control.

The study presented limitations in relation to the previous data regarding the number of employees in the co-operatives, due to there not being an official reference on behalf of the Popular and Solidary Economy Superintendent of the Government of Ecuador. As such, it was necessary to work with an approximate standard provided by the Ministry of Labor in this country. Finally, for future lines of investigation, we propose the application of this measuring instrument to other business groups with the purpose of ratifying the signaled adaptability and, in addition, analyzing whether there is a relationship among internal marketing and other variables of human capital management, such as, for example, the organizational commitment. Finally, a more in-depth study of the causal relationship between the dimensions of internal marketing and the co-creation of value in these institutions of social economy would be interesting.

## Data Availability Statement

The raw data supporting the conclusions of this article will be made available by the authors, without undue reservation.

## Ethics Statement

Ethical review and approval was not required for the study on human participants in accordance with the local legislation and institutional requirements. Written informed consent for participation was not required for this study in accordance with the national legislation and the institutional requirements.

## Author Contributions

FG, NM, ML, and AH conceptualized the work and reviewed the literature, interpreted and curated the data, and wrote the manuscript. The authors read and revised the manuscript several times. All authors contributed to the article and approved the submitted version.

### Conflict of Interest

The authors declare that the research was conducted in the absence of any commercial or financial relationships that could be construed as a potential conflict of interest.
